# Adaptation of the Food Choice Questionnaire: the case of Hungary

**DOI:** 10.1108/BFJ-07-2017-0404

**Published:** 2018-07-02

**Authors:** Zoltán Szakály, Enikő Kontor, Sándor Kovács, József Popp, Károly Pető, Zsolt Polereczki

**Affiliations:** 1 Institute of Marketing and Commerce, Faculty of Economics and Business, University of Debrecen, Debrecen, Hungary; 2 Institute of Sectorial Economics and Methodology, Faculty of Economics and Business, University of Debrecen, Debrecen, Hungary; 3 Institute of Rural Development, Tourism and Sport Management, Faculty of Economics and Business, University of Debrecen, Debrecen, Hungary

**Keywords:** Validity, Motives, Food choice, Questionnaire

## Abstract

**Purpose:**

The purpose of this paper is to examine the applicability of the original 36-item Food Choice Questionnaire (FCQ) model developed by Steptoe *et al.* (1995) in Hungary.

**Design/methodology/approach:**

The national representative questionnaire involved 1,050 individuals in Hungary in 2015. Several multivariable statistical techniques were applied for the analysis of the data: confirmatory factor analysis, principal component analysis, and cluster and Log-linear analysis.

**Findings:**

The results indicate that the original nine-factor model is only partially applicable to Hungary. This study successfully managed to distinguish the following factors: health and natural content, mood, preparation convenience, price and purchase convenience, sensory appeal, familiarity, and ethical concern. The FCQ scales proved to be suitable for the description of clusters based on specific food choices and demographic characteristics. By using the factors, the following five clusters were identified: modern food enthusiast, tradition-oriented, optimizer, easy-choice and un-concerned, all of which could be addressed by public health policy with individually tailored messages.

**Originality/value:**

The Hungarian testing process of the FCQ model contributes to an examination of its usability and provides the possibility of fitting the model to different cultures.

## 1. Introduction

Food choices can be described as a complex process, whose influencing factors can be divided into two categories: internal (food) effects (e.g. sensory aspects) and external (non-food) effects (e.g. psychological, social and cultural factors) (Eertmans *et al.*, 2005). Attempts have been made to describe and approach these factors from different perspectives.

The literature draws attention to the fact that consumer behavior is changing, and a kind of nutritional revolution is occurring. The “new” consumer builds on trust and takes on role as a conscious innovator and opinion leader (Lewis and Bridger, 2001). Food purchases by individuals are increasingly driven by information, attitudes and other complex psychological factors and are decreasingly influenced by price and income. Lifestyle changes can also be observed. Consumers’ health and environmental awareness is steadily growing with the increasing availability of information. Research by Schor (1999) suggests that the eating habits of consumers have been influenced by social status and expectations. In addition, a trend toward sustainable consumption has appeared. The public’s interest in sustainability is steadily increasing and attitudes are generally positive, but consumer behavior is not always consistent with this belief (Kearny, 2010).

Globalization has changed consumers’ food consumption culture with its impact on food production, purchasing and distribution. The growth in income, trade liberalization, transnational corporations, and the role of the media and marketing are among the drivers of food consumption. In most developed countries, increasing income has beneficial effects, which are reflected in better nutrition and better health. On the one hand, the liberalization of trade has also brought about a qualitative improvement in food quality standards and safety, while on the other hand it has increased the availability of cheap, highly processed low-health foods (Popkin, 2006; Hawkes, 2007; Hawkesworth *et al.*, 2010; Kearny, 2010). Different members of the food supply chain are trying to identify and satisfy the changed needs with better processed and higher added-value products. Ready to cook and ready-to-eat foods are becoming more popular. Furthermore, the need for comfort and quality have, for example, induced packaging technology changes that are adapted to the decreasing size of households and ageing societies. The foodservice industry has reacted with away-from-home food products to the market trend toward out-of-home meals (Davis and Stewart, 2002).

The proliferation of transnational corporations has also emphasized the role of food marketing, which basically determines food trends and consumption patterns. The mistrust between the media and the food industry also fundamentally affects consumers’ beliefs related to food, and consequently the choices they make (Anderson, 2000). In addition, it is important to note that according to research, the above-mentioned external factors can also alter sensory perceptions (Lee *et al.*, 2013).

Steptoe *et al.* (1995) have developed a multi-dimensional measure of the motives underlying the selection of food. This Food Choice Questionnaire (FCQ) has provided a tool to simultaneously assess the relative importance of different factors in food choice (Steptoe *et al.*, 1995). The original FCQ comprises 36 statements, representing health and non-health-related food attributes, and nine factors were identified that confirm the motivations behind our food choices: health, mood, convenience, sensory appeal, natural content, price, weight control, familiarity and ethical concern. Each of these factors includes three to six items. When carrying out the tests participants were asked to endorse the statement: “It is important to me that the food I eat on a typical day […]” for each of the 36 items, by scoring on a four-point scale, which ranged from 1=“Not at all important” to 4=“Very important.”

In their UK samples, the results of Steptoe *et al.* (1995) showed that sensory appeal, health, convenience and price were the most important motives, but there were differences in the food choice motives associated with gender, age and income.

The original FCQ–or modified versions of it–has been applied in many different ways and in many different countries over the last 25 years. Regarding the item structure of the FCQ, Lindeman and Väänänen (2000) in Finland developed three new scales for measuring ethical food choice motives: ecological welfare (the subscales being animal welfare and environmental protection), political values and religion. Rahman *et al.* (2013) included additional items related to religion and risk perception in Malaysia, Kornelis *et al.* (2010) applied complementary scales that assess non-food information in a Dutch sample and Roos *et al.* (2012) also used the modified FCQ to examine parental family food choice motives in Finland. Furthermore, the adapted FCQ model has been implemented by many other researchers (Lockie *et al.*, 2002; Ares and Gámbaro, 2007; Scheibehenne *et al.*, 2007; Pieniak *et al.*, 2009, 2013; Honkanen and Frewer, 2009; Share and Stewart-Knox, 2012; Crossley and Nazir, 2002; Dowd and Burke, 2013; Pula *et al.*, 2014; Gagić *et al.*, 2014; Ooi *et al.*, 2015; Dikmen *et al.*, 2016). The structure of factors has also varied from study to study. For example, in Ooi *et al.*’s (2015) Malay adolescent sample, the new factors were religion, parents, peers and media, and in Pula *et al.*’s (2014) UK study, a new impression management factor was included.

Since the initial use of this instrument, it has been adapted to include different related areas such as traditional foods (Pieniak *et al.*, 2009, 2013), functional foods (Ares and Gámbaro, 2007), organic foods (Lockie *et al.*, 2002) diet behavior (Pollard *et al.*, 1998), food neophobia (Eertmans *et al.*, 2005), food risk perception (Rahman *et al.*, 2013) and “sustainably sourced food” (Dowd and Burke, 2013).

As can be seen above, the FCQ has been applied at the national level, but there is also much interest in testing the cross-cultural validity of the FCQ model (Prescott *et al.*, 2002; Eertmans *et al.*, 2006; Pieniak *et al.*, 2009; Milošević *et al.*, 2012; Januszewska *et al.*, 2011; Markovina *et al.*, 2015). Prescott *et al.* (2002) was one of the first to apply the FCQ in Asia, and their results indicated a strong relationship between food choice motives and nationality. More recently, the measurement invariance has been demonstrated by Pieniak *et al.* (2009) across six countries, by Januszewska *et al.* (2011) across four countries and by Markovina *et al.* (2015) across nine European countries. In contrast to these findings, Milošević *et al.*’s (2012) analysis indicated that the original nine-factor design displayed a suboptimal fit for the Western Balkan Countries. Furthermore, Eertmans *et al.*’s (2006) results of the test across three countries (Canada, Belgium and Italy) did not support the generalizability of the FCQ’s factor structure. They suggested that its items and construct may have different connotations across western urban populations, whether in English-speaking or non-English-speaking countries. Fotopoulos *et al.* (2009) also did not reinforce the robustness of the original FCQ. There have been some psychometric problems, but in their opinion, at the subpopulation level the original FCQ can form the basis for a new typology.

When analyzing the data, answers to three main questions were sought:

1. Can the original FCQ be applied to this sample?
2. If the original model cannot be applied, how are the factors modified?
3. Can the FCQ model be used to create individual consumer segments based on consumers’ food choice habits?

## 2. Materials and methods

### Sampling

The data collection was carried out in November 2015 with the help of a market research firm. The sample consisted of 1,050 individuals. For sample size calculation, we used the following formulas:

$$\mathrm{SS}=\frac{Z^{2}\cdot p\cdot \left(1-p\right)}{{\mathrm{err}}^{2}}\;\mathrm{and}\;\mathrm{corrected}\;\mathrm{SS}=\frac{\mathrm{ss}}{1+\left(\left(\mathrm{ss}-1\right)/\mathrm{population}\right)},$$

where *Z* is the *Z*-value (e.g. 1.96 for 95% confidence level), *p* is the percentage picking a choice in a question and err is the margin of error or confidence level. In this formula, we used 8,000,000 for the population, *Z*=1.96 for 95% confidence level, the error rate was 3 percent, for the worst case scenario, *p*=0.5 SS was 1,067 and for *p*=0.4 SS was 1,024, so we set the sample size to 1,050.

Representativeness for regions and for types of settlement was ensured by the applied quoted sampling method. The sample pattern met the quotas previously defined by the Hungarian Central Statistical Office. In the assigned settlements a random walking method was used to ensure total randomness in selection. In the second step, the interviewed individual within a household/family was selected by using the so-called birthday-key. With this method, randomness was ensured in the second step as well. (The reason for the involvement of young people under the age of 18 was that young people, in the majority of cases, make independent purchasing decisions and their influence on the family’s purchases of food is extremely high).

Since random walking does not ensure the sample is a reflection of the entirety of the population (the number of the female and elderly respondents was higher than the national average), the sample of the people was corrected by multi-dimensional weighting factors (gender and age). After these methods were applied, the sample was representative of the structure of the Hungarian population in all the three aspects (region of residence, gender and age).

We took care that the personal data of the interviewed individuals enjoyed complete protection and anonymity. The data were collected through a standard questionnaire. The questionnaire used in this research was completed after asking several individuals from the target group (pre-testing phase).

### Questionnaire

In the analysis, the 36-item questions of the original FCQ with a five-point Likert scale were used. An answer of 1 represented “I do not take this into account at all,” while a 5 represented “I fully take this into account.” The distribution of the sample is shown in Table I.

### Data analysis

Several multivariable statistical techniques were applied for the analysis of the data: confirmatory factor analysis (CFA), principal component analysis (PCA) and cluster analysis. As a first step, a CFA was applied and a fit estimated for Steptoe *et al.*’s (1995) nine independent factor FCQ model for Hungary. Goodness of fit was assessed using a variety of measures.

A highly significant difference was found between the original and the expected covariance structure according to the *χ*^2^ indices, which indicated there was a large unexplained part in the original model (*χ*^2^(630)=16,798.2; *p*<0.001). The Tucker Lewis index also lay outside the conventional acceptance limits of 0.95. Although the RMSEA value of 0.065 indicated a good fit, as it was below the conventional value of 0.08, CFA goodness-of-fit measures suggested that the original factor structure cannot be applied directly to the Hungarian data, and therefore explanatory factor analysis should be followed (Linting *et al.*, 2007; Fotopoulos *et al.*, 2009). PCA was employed with varimax rotation to correctly identify the independent principal components (PCs). In order to detect the internal consistency of the PCs and measure scale and subscale reliability, Cronbach’s *α* was calculated for each subscale and for the whole scale. The identified dimensions have a reliability above the recommended 0.6–0.7, and all the component loadings were greater than or equal to 0.4–0.5, while the extracted variance was around 0.7 (Hair *et al.*, 1998). The assessment of statistically significant differences across the PCs was established by analysis of variance (ANOVA) and an independent sample *t*-test. A significance level of 5 percent was used for evaluating the results. The Levene’s test was also applied to check homogeneity and found the studied groups to be homogenous in terms of their standard deviation. In order to measure the strength of the relationships, effect sizes and the 95% confidence intervals for the difference between subsets were reported.

Finally, cluster analysis was performed using the standardized factor scores from the PCA. The following clustering methods were used and compared: hierarchical clustering with Euclidean distance (using simple, complete and average linkage, centroid, median and Ward’s method) and K-means clustering (using MacQueen’s method). The above-mentioned clustering algorithms were performed with different numbers of clusters 3–6. The following clustering quality indices were used to determine the proper clustering algorithm and to establish the suitable number of clusters: the Silhouette index (Chen *et al.*, 2002), the Calinski-Harabasz index (Zhao and Karypis, 2005; Shu *et al.*, 2003) and the Dunn index (Bolshakova and Azuaje, 2003). For all indices, the highest value indicates the most appropriate cluster configuration. Based on the applied indices, K-means clustering with five clusters proved to be the proper configuration as it resulted in the highest quality indices. In order to validate the adaptive FCQ clusters, a leave-one-out (LOO) validation technique was used. During the LOO validation, each FCQ factor was omitted one by one and the stability of the cluster structure was examined. The established FCQ clusters proved to be stable during the validation. In the case of scale profile variables, an ANOVA test was applied to compare average cluster scores.

Furthermore, categorical data were analyzed by log-linear analysis. The relationship between four factors (FCQ clusters, age, gender, education level) was investigated. If two factors are not independent of each other, then an interaction effect exists between them. The saturated model contains all possible interactions between the studied factors while the restricted model contains only some of the interaction effects. Log-linear analysis uses Pearson *χ*^2^ statistics in which the restricted model is compared to the saturated model. The null hypothesis is that there is no difference between the two models; therefore, the lack of difference (*p*>0.05) indicates a good model fit. Among all possible interactions, the following conditional independence model could be fitted to our data:

U=Constantterm+FCQcluster×Gender+FCQcluster×Age+FCQclusterbyEducationlevel,

where *U* denotes the logarithm of the observed frequencies and × denotes interaction effect.

The *R* 3.0.3 statistical software package was used for all analyses, the clusterCrit package was used for measuring the cluster quality and the CFA was calculated by the LAVAAN package (LAtent VAriable ANalysis), while log-linear analysis was performed using loglm function in the MASS package.

## 3. Results

### Food choice

Since the nine-factor model which derives from the original FCQ statements is not appropriate for the sample, PCA analysis was used to discover what modifications occurred in the original model’s factors (Table II).

As can be seen from the results, the original model can only be partially applied to this sample. Three other factors were listed among the statements. Of the original factors only sensory appeal, mood, familiarity and ethical concern remained in their original form. From the convenience factor, the items referring to ease of obtaining the food moved to the price factor, and together formed the price and purchase convenience factor. Health and natural content, and weight control, which were originally separate factors, entered the new model as a single factor. The new factor structure is shown in Table III.

According to our results, seven factors could be identified. These were health and natural content, mood, preparation convenience, price, sensory appeal, familiarity and ethical concerns.

If we also examine the results according to their relationships with the background variables, significant differences can be noticed in several cases. In terms of gender difference, the *t*-test shows differences in the following factors: health and natural content (*t*(1,048)=−6.3; *p*<0,001, effect size=0.39; mean difference=−0.38 in 95% CI of −0.50 and −0.26), price and purchase convenience (*t*(1,048)=−2.2; *p*=0,028, effect size=0.24; mean difference=−0.13 in 95% CI of −0.26 and −0.02) and familiarity (*t*(1,048)=2.5; *p*=0,012, effect size=0.25; mean difference=0.15 in 95% CI of 0.03 and 0.28). Health and naturalness is relatively more important for women (mean score=0.18 vs −0.20) when choosing food, as well as price and ease of purchase (mean score=0.06 vs −0.07). For men, however, it is relatively more important that the food they choose is familiar to them (mean score=0.08 vs −0.07). The ANOVA test revealed significant differences in terms of age group for the following factors: mood (*F*(5,1043)=5.8; *p*<0.001, effect size=0.61; mean score range=0.47), price and purchase convenience (*F*(5,1043)=5.9; *p*<0.001, effect size=0.61; mean score range=0.56), preparation convenience (*F*(5,1043)=4.2; *p*=0.001, effect size=0.51; mean score range=0.39) and ethical concerns (*F*(5,1043)=3.7; *p*=0.002, effect size=0.63; mean score range=0.55).

Mood and preparation convenience were the decisive factors for the younger age group (under 30), while price had a relatively important role for the older age group, and was less important for younger people. Ethical questions appeared as a decisive factor among middle-aged people (aged 30–60). The familiarity of the food was important for those over 50. In terms of the level of schooling, in the ANOVA tests almost all the FCQ components showed differences: (familiarity–*F*(3,1045)=5.9; *p*=0.001, effect size=0.60; health and natural content–*F*(3,1045)=30.7; *p*<0.001, effect size=0.62; mood–*F*(3,1045)=5.0; *p*=0.002, effect size=0.61; price and purchasing convenience–*F*(3,1045)=6.2; *p*<0.001, effect size=0.60; preparing convenience–*F*(3,1045)=3.0; *p*=0.028, effect size=0.70; and ethical concerns–*F*(3,1045)=5.1; *p*=0.002, effect size=0.68).

To conclude this stage of the investigation, clusters were developed from the seven factors which had emerged previously. The results are shown in Table IV.

From the seven factors, five clusters were created. With the first cluster (modern food enthusiast), factors 2, 5 and 3 were decisive. With the second cluster (tradition-oriented) it was factor 6, with the third cluster (optimizer) factors 4 and 5 and with cluster 4 (easy-choice) factors 6 and 3. With the fifth cluster (un-concerned) negatively valued factors appeared.

In order to better understand the internal structure of clusters, log-linear analysis was performed on the demographic background variables. The results are shown in Table V regarding age and education levels. The Pearson *χ*^2^ value (*χ*^2^(df=144)=167.05) indicated an acceptable model fit (*p*=0.092).

## 4. Discussion

### FCQ structure

The first two questions of our research attempted to discover whether the original FCQ construction could be applied to our sample and, if not, how the structure of the factors could be modified. The nine-factor FCQ construction developed by Steptoe *et al.* (1995) has been widely used to investigate motivations behind the choice of foods. Later on, Lindeman and Väänänen (2000) extended the range of aspects with three ethical factors. During the widespread application of this measuring methodology several authors, such as Prescott *et al.* (2002), and Januszewska *et al.* (2011) demonstrated measurement invariance cross-culturally, while other authors’ results did not support the generalizability of the FCQ’s factor structure (these included Eertmans *et al.*, 2006; Fotopoulos *et al.*, 2009; Pula *et al.*, 2014).

The factor structures which were developed during the examinations ranged from 5 to 13 factors, of varying content. As a result of our representative survey, it is established that the original nine-factor model is only partially applicable to the Hungarian sample. On the basis of our results, seven factors were distinguished, namely health and natural content (+ weight control), mood, preparation convenience, price and purchase convenience, sensory appeal, familiarity and ethical concerns. Comparing these to the results of other researchers, there is a clear tendency for the health and natural content (as well as, in some cases, weight control) factors to merge together (see Eertmans *et al.*, 2006; Milošević *et al.*, 2012; Ooi *et al.*, 2015; among others). At the same time, another characteristic can also be observed, in which the convenience factor becomes divided into purchase and preparation convenience. Our results also support this, since the purchase convenience, when added to price, creates one factor, while preparation convenience is its own independent factor. This is also apparent in the work of Eertmans *et al.* (2006), Ares and Gámbaro (2007), Milošević *et al.* (2012), Gagić *et al.* (2014) and Ooi *et al.* (2015).

Regarding the order of importance of the various factors, the Hungarian sample order agrees with research studies that place sensory appeal, price and purchase convenience, and preparation convenience factors at the top of the list. (The average values of the individual factors are shown in Table III.) Similar preferences are reported with British (Steptoe *et al.*, 1995) and Russian (Honkanen and Frewer, 2009) consumers as regards motivating factors in the choice of food.

Moreover, the partial results of Januszewska *et al.* (2011), according to which for Hungarian consumers the health factor is less important, confirm this, unlike, for example, the full samples on the same research, and unlike the evaluations of Steptoe *et al.*’s (1995) British sample, or Gagić *et al.*’s (2014) Serbian sample, where consumers consider health to be the second most essential aspect. At the same time, in our research Familiarity occupies a high, fourth place, while in other cultures it is considered to be among the least important. It seems that for Hungarian consumers (and, as expected, most particularly men) the fact that food is familiar is much more important, and they are much less open to novelty. However, for Hungarians ethical questions are also less important.

Analysis on the basis of gender revealed that three of the FCQ seven factors showed a significant difference. The three factors are health and natural content, price and purchase convenience, and familiarity. For women, health and natural content (weight control) was relatively more important, as well as price and purchase convenience, while for men familiarity proved to be the most essential. Compared to other FCQ measures, differences between the genders in the evaluation of the factors listed above also appear in the work of other researchers. The importance of the health factor among women respondents was noted in the original research of Steptoe *et al.* (1995). At the same time, Crossley and Nazir’s (2002) results showed that among dental students, women were significantly more aware of–among other things–the health and convenience aspects when they were choosing food. The differences are also confirmed in the research carried out by Januszewska *et al.* (2011), in which women over the whole sample gave more importance to natural content and weight control factors.

At the same time, there are research studies which have not found gender differences in the factors (e.g. Milošević *et al.*, 2012). Although Gagić *et al.*’s (2014) study also showed that there is almost no gender difference between the importance of the factors, women–just as in our study–evaluate convenience higher than men (although this refers to the original convenience factor). In their work, familiarity shows no significant difference, unlike in our research.

If we examine the order of the factors in terms of another background variable, age group, it becomes clear that the mood, price and purchase convenience, preparation convenience and ethical concerns factors show differences. Among these factors, it is ethical concerns which shows agreement with the age group-based results obtained by Steptoe *et al.* (1995). Similarly to the present research results, Prescott *et al.* (2002) found that it was the older consumers who evaluated the ethical concern and familiarity factors more highly than the young. However, the mood factor in our study was decisive in the under 30 age group, while in Prescott *et al.* (2002) it was more significant for older consumers. Rahman *et al.* (2013)–similarly to our results–found age to have a significant negative correlation with mood (i.e. the older a person becomes, the less important this factor is for them).

On the basis of our results, it can be stated that in almost all factors there was a difference in terms of level of education, and compared to other background variables, it had a greater effect. Steptoe and Wardle (1999) showed differences based on educational status, but only in the case of price, familiarity, mood and sensory appeal factors. The evaluations made by those with lower educational qualifications, however, agree with those in our research, given that they gave great emphasis to price and familiarity factors. In this research, however, the greatest effect was observed in the cases of ethical concerns (for those with high-level qualifications), and the preparation convenience (for those with lower-level qualifications).

### FCQ clusters

During the analysis, five clusters were created from the seven FCQ factors on the basis of differences in motivational preferences.

In the case of Cluster 1, called the “Modern food enthusiast” group, the mood, and the sensory characteristics were the most important factors, but the ease of preparation factor also had an effect. Based on the analysis, men and young people (from 14 to 29 years of age), and people with higher and secondary education are more likely to belong to Cluster 1 than to other clusters. In fact, in terms of age group they represent the *y* and *z* generations. First, this age group cannot yet earn a living for themselves, so the primary criterion in the decision-making process is the current mood, or sensory appeal; second, this age group represents young adults (men) for whom long meal preparations are not attractive, but who expect a consistent sensory quality. In addition, this age group is most affected by the daily stress and challenges of life, and the food and meals consumed may actually help to combat stress and relieve tension.

In Cluster 2 or the “Tradition-oriented” group, as is clear from the name, consumers prefer to choose foods which they are familiar with. It is mainly men and middle-aged people (from 30 to 59 years of age) with higher and secondary education who are more likely to belong to Cluster 2. The analysis of the FCQ factors also showed that familiarity is a particularly important factor for Hungarian customers, especially for men. It probably gives a sense of stability, and perhaps also assists them in making speedy choices, and therefore means not much cognitive capacity is required when purchasing food. It is noteworthy that this is the cluster where consumers–if only slightly–also take ethical aspects into account. Familiarity perhaps also represents a kind of guarantee of the place of origin.

Naturally, a price-oriented group emerged as well, the Cluster 3 group, referred to as “Optimizer,” although this group cannot be termed particularly price-sensitive, since it was also important for them that the product had the right taste, aroma, texture and appearance. It is mainly middle-aged, and older women who belong to Cluster 3 (the proportion of consumers over 60 is significant). With regard to educational qualifications, there is no outstanding category. The cluster features reinforce the fact that the price sensitivity and purchase convenience is the most important motive for women, for whom the sensory/gastronomic appeal of the food is just as important.

Cluster 4 included those who experienced a feeling of pleasure when purchasing food, the “Easy-choice” group. For them the familiarity of the food and its ease of preparation were important, but sometimes they were influenced by mood when making a choice. The likelihood of belonging to this cluster is almost equal in terms of gender distribution. Young people with lower qualifications are more likely to be included in this cluster.

The Cluster 5 group was termed the “Un-concerned,” since we could not identify any decisive factor in their case; they approach the task of choosing and buying food with indifference, and do it as a routine. Older women (over the age of 60) are more likely to be members of this cluster as well as those with lower–i.e. vocational and primary school–educational qualifications.

## 5. Conclusions

During the examination, we sought answers to the three questions that were presented in the Introduction.

Regarding the first and second questions (can the original FCQ be applied to the sample?–if the original model is not applicable, how can the factors be modified?), our conclusion was that the original FCQ model, with its 36 statements, cannot in its entirety be applied to the sample, although the differences experienced were minimal. Instead of the original nine factors, seven factors were identified in the sample. The study considered the reason for these differences to lie in cultural differences.

Another conclusion of the study is that the FCQ scales–although on a different factor structure–have also proved in the Hungarian sample that food choices and demographic characteristics can be described as clusters. These clusters provide an opportunity for public health policy to influence–on appropriate platforms and with target group-specific messages–the food choice and consumption habits of the Hungarian consumer. Taking into account the fact that factors related to health claims, natural ingredients and ethical considerations did not show an outstanding value in a single cluster, a clear strategic direction for public health and nutrition policy is evident.

The results show that there are seven factors simultaneously present in the mindset of the Hungarian population. Accordingly, companies and public health organizations can tailor their media messages in a customized way, according to the way consumers think. The main messages are best formulated along the lines of health and naturalness, a good state of mind, food preparation comfort, price sensitivity and convenient availability.

The research also identified those target groups who could be targeted with messages in a differentiated way. For the modern food enthusiast group, the media message can be formulated along lines of good mood and enjoyment, while for the tradition-oriented cluster it can focus on intimacy and ethical considerations. For the optimizer, price, comfort and enjoyment can be an effective argument, while in the Easy-choice cluster all aspects that make it easily available are important. Last but not least, targeting and reaching the Un-concerned is doubtful as this group has no single value, except for the acceptance of favorable prices. Based on the results of the research, public health organizations have the opportunity to influence the health of the Hungarian population in a positive direction, an important element of which is the emphasis on conscious food choice.

## Figures and Tables

**Table I:** Distribution of the sample according to the most important background variables

	Sample distribution		Census proportions^a^
Name	Individuals	%	%
*All respondents*			
Total	1,050	100.0	
*By gender*			
Men	494	47.0	46.9
Women	556	53.0	53.1
*By age (years)*			
14–18	65	6.2	6.6
19–29	164	15.6	15.8
30–39	187	17.8	18.4
40–49	175	16.7	15.3
50–59	164	15.7	16.7
60 and older	294	28.0	27.2
*By highest educational qualification achieved*			
Up to 8 years of schooling	164	15.6	31.6
Vocational or specialist school	334	31.8	21.3
High school qualification	370	35.2	30.1
Higher education degree	183	17.4	17.0
*By region*			
Central Hungary	133	12.6	12.6
Central Transdanubia	117	11.2	11.3
Western Transdanubia	106	10.1	10.3
Southern Transdanubia	99	9.4	10.0
Northern Hungary	120	11.4	11.9
Northern Great Plain	149	14.2	12.4
Southern Great Plain	140	13.3	13.1
Budapest	186	17.8	18.4

**Note:** ^a^On the basis of data from the 2011 census; the census data distribution also shows the 14-year old or older age group

**Table II:** Results from the principal component analysis

Item number	Original factors	Extracted factors	Items on other factors than the original
	*1. Health*	1 and 5 and 7 health, natural content	
22	Contains a lot of vitamins and minerals	0.76	
29	Keeps me healthy	0.70	
10	Is nutritious	0.40	
27	Is high in protein	0.68	
30	Is good for my skin/teeth/hair/nails	0.67	
9	Is high in ﬁber and roughage	0.74	
	*2. Mood*		
16	Helps me cope with stress	0.73	
34	Helps me cope with life	0.72	
26	Helps me relax	0.79	
24	Keeps me awake/alert	0.72	
13	Cheers me up	0.69	
31	Makes me feel good		Sensory appeal 0.62
	*3. Convenience*	3.1 Preparation convenience	
1	Is easy to prepare	0.83	
15	Can be cooked very simply	0.77	
28	Takes no time to prepare	0.83	
35	Can be bought in shops close to where I live or work		Price and purchase convenience 0.60
11	Is easily available in shops and supermarkets		Price and purchase convenience 0.57
	*4. Sensory appeal*		
14	Smells nice	0.69	
25	Looks nice	0.75	
18	Has a pleasant texture	0.75	
4	Tastes good	0.41	
2	*5. Natural content contains no additives*	1 and 5 and 7 health, natural content 0.67	
5	Contains natural ingredients	0.67	
23	Contains no artiﬁcial ingredients	0.69	
	*6. Price*	6 price and 3.2 purchase convenience	
6	Is not expensive	0.80	
36	Is cheap	0.83	
12	Is good value for money	0.61	
	*7. Weight control*	1 and 5 and 7 health, natural content	
3	Is low in calories	0.71	
17	Helps me control my weight	0.66	
7	Is low in fat	0.72	
	*8. Familiarity*		
33	Is what I usually eat	0.56	
8	Is familiar	0.40	
21	Is like the food I ate when I was a child	0.73	
	*9. Ethical concern*		
20	Comes from countries I approve of politically	0.65	
32	Has the country of origin clearly marked	0.69	
19	Is packaged in an environmentally friendly way	0.45	

**Note:** PCA loadings on numbered items (*n*=1,050)

**Table III:** Descriptive statistics and reliabilities of the original FCQ

Item number	It is important to me that the food I eat on a typical day	Median	IQR
*1. Health and natural content*		3.52	2.18
22	Contains a lot of vitamins and minerals	3.74	1.96
29	Keeps me healthy	3.96	1.69
10	Is nutritious	4.45	1.35
27	Is high in protein	3.11	2.13
30	Is good for my skin/teeth/hair/nails	2.94	2.40
9	Is high in ﬁber and roughage	3.38	2.00
2	Contains no additives	3.62	2.14
5	Contains natural ingredients	3.96	1.76
23	Contains no artiﬁcial ingredients	3.86	2.00
3	Is low in calories	2.66	2.19
17	Helps me control my weight	2.87	2.40
7	Is low in fat	3.15	1.95
31	Makes me feel good	4.20	1.69
	Cronbach’s *α*: 0.90		
*2. Mood*		2.72	2.44
16	Helps me cope with stress	2.21	2.42
34	Helps me cope with life	2.56	2.55
26	Helps me relax	2.55	2.47
24	Keeps me awake/alert	2.06	2.41
13	Cheers me up	3.21	2.69
	Cronbach’s *α*: 0.82		
*3. Preparation convenience*		4.03	1.79
1	Is easy to prepare	4.07	1.80
15	Can be cooked very simply	4.19	1.68
28	Takes no time to prepare	3.65	1.96
	Cronbach’s *α*: 0.83		
*4. Price and purchase convenience*		4.36	1.52
6	Is not expensive	4.26	1.69
36	Is cheap	4.13	1.60
12	Is good value for money	4.62	0.94
35	Can be bought in shops close to where I live or work	4.29	1.62
11	Is easily available in shops and supermarkets	4.40	1.45
	Cronbach’s *α*: 0.78		
*5. Sensory appeal*		4.42	1.43
14	Smells nice	4.35	1.56
25	Looks nice	4.43	1.37
18	Has a pleasant texture	4.24	1.66
4	Tastes good	4.74	0.80
	Cronbach’s *α*: 0.65		
*6. Familiarity*		3.86	1.76
33	Is what I usually eat	3.92	1.62
8	Is familiar	4.15	1.61
21	Is like the food I ate when I was a child	3.44	2.10
	Cronbach’s *α*: 0.71		
*7. Ethical concern*		2.43	2.49
20	Comes from countries I approve of politically	1.53	1.95
32	Has the country of origin clearly marked	2.99	2.70
19	Is packaged in an environmentally friendly way Cronbach’s Alpha: 0.67	2.79	2.11
	Cronbach’s *α*: 0.71. mean: 3.47		

**Notes:** *n*=1,050. Five-point Likert scale was used

**Table IV:** Characteristics of the FCQ clusters

	Average factor scores by cluster					
	FCQ clusters					
	1	2	3	4	5	
Factors	Modern food enthusiast (*n*=121)	Tradition-oriented (*n*=140)	Optimizer (*n*=206, 207)	Easy-choice (*n*=360)	Un-concerned (*n*=223)	*F*-test^a^
Factor 1 (health and natural content)	−0.62	0.12	−0.12	0.27	−0.07	21.2
Factor 2 (mood)	0.46	−0.63	−0.40	0.39	−0.13	51.7
Factor 3 (prep. convenience)	0.30	−0.81	−0.48	0.47	0.03	73.7
Factor 4 (price and purchase convenience)	−1.19	−1.01	0.59	0.30	0.24	181.5
Factor 5 (sensory appeal)	0.36	0.07	0.60	0.34	−1.35	275.2
Factor 6 (familiarity)	−0.87	0.72	−0.73	0.58	−0.24	166.1
Factor 7 (ethical concern)	−0.55	0.35	0.33	−0.01	−0.21	23.3

**Notes:** *n*=1,050. ^a^The *F*-tests are for descriptive purposes only. All F values are significant at the 1% level

**Table V:** Parameter estimates of the education/age and FCQ cluster effect

FCQ cluster	1. Modern food enthusiast	2. Tradition-oriented	3. Optimizer	4. Easy-choice	5. Un-concerned
*Parameter estimates of education level*					
Higher educated	0.173	0.133	0.012	−0.125	−0.193
Secondary school	0.113	0.220	−0.078	−0.087	−0.147
Vocational school	−0.380	−0.203	0.090	0.193	0.298
Primary school	0.092	−0.150	−0.025	0.020	0.042
Odds (higher vs primary)	1.08	1.33	1.04	0.87	0.79
*Parameter estimates of the age*					
14–18	0.451	0.092	−0.048	0.071	−0.566
19–29	0.243	−0.210	−0.141	0.016	0.091
30–39	0.168	0.167	−0.254	−0.237	0.157
40–49	0.113	0.036	0.057	−0.043	−0.162
50–59	−0.588	0.090	0.150	0.236	0.110
60<	−0.387	−0.175	0.235	−0.428	0.369
Odds (14–18 vs 60<)	2.31	1.31	0.75	1.65	0.39

**Note:** *n*=1,050
